# Socio-demographic characteristics and clinical profile among suicide attempters

**DOI:** 10.1192/j.eurpsy.2023.1850

**Published:** 2023-07-19

**Authors:** R. Ouali, R. Masmoudi, F. Guermazi, F. cherif, I. Feki, O. Chakroun, E. Derbel, R. sellami, J. Masmoudi, N. Rekik

**Affiliations:** 1 Psychiatry A, hôpital hedi chaker; 2 Emergency Department, Habib Bourguiba University Hospital; 3 Psychiatry A, Hedi Chaker University Hospital, sfax, Tunisia

## Abstract

**Introduction:**

Suicide is a real public health problem. Like many other countries, Tunisia seems to be experiencing an amplification of the phenomenon. Suicide attempts are much more frequent and are estimated to be around 20 times the number of suicides

**Objectives:**

The objective was to describe the sociodemographic characteristics and clinical profile of suicide attempters in patients hospitalized in the emergency room.

**Methods:**

This study was carried out with patients admitted to vital emergencies for attempted suicide over a period of 6 months.

A pre-made questionnaire was used to collect sociodemographic and clinical data. We used the SIS “suicide intention scale” to assess the intent of the suicide attempt and the PHQ9 “PATIENT HEALTH QUESTIONNAIRE” to assess the presence and severity of depressive symptoms.

We excluded Patients with major cognitive impairment, which prevents understanding of the questionnaire.

**Results:**

Our sample consisted of 101 patients. Of the participants, 69.3% were female. Their age varied between 18 and 65 years with an average age of 30.93 years. The socioeconomic level was low in 23.8% of cases. The level of education did not exceed secondary school for 91% of suicides. Almost half of suicide attempters (45%) have been professionally inactive. Participants included in our study were single in 51.5% of cases. Participants had a family history of attempted suicide in 15% of cases. Prior psychiatric follow-up was found in 34% of suicides attempts. More than a third (36.6%) of participants had moderate to severe depression according to the results of the PHQ-9. Recurrences concerned 44% of suicides attempt in our survey and the average number of previous suicides attempts was 3.9. Suicidal intent was rated strong in 47% of suicides attempts .

**Conclusions:**

Suicidal behavior is one of the leading causes of death and disability worldwide. In our study, more than 1 in 3 suicide attempters had depression. Further research is needed to identify suicide risk factors and to examine the relationship between the presence of mental illness and suicidal attempt

**Disclosure of Interest:**

None Declared

